# Establishing a mathematical standard model for calculating the volume of peripheral arteriovenous synchronous blood exchange transfusion in neonates with severe hyperbilirubinemia

**DOI:** 10.12669/pjms.41.8.12393

**Published:** 2025-08

**Authors:** Sheng Cheng, Jingjing Pan, Bing Wang, Tongjin Yin

**Affiliations:** 1 Sheng Cheng Department of Pediatrics, Affiliated Hospital 6 of Nantong University; Pediatrics Department of Yancheng Third People’s Hospital, Yancheng, Jiangsu Province 224000, P.R. China; 2 Jingjing Pan Department of Pediatrics, Affiliated Hospital 6 of Nantong University; Pediatrics Department of Yancheng Third People’s Hospital, Yancheng, Jiangsu Province 224000, P.R. China; 3 Bing Wang Department of Pediatrics, Affiliated Hospital 6 of Nantong University; Pediatrics Department of Yancheng Third People’s Hospital, Yancheng, Jiangsu Province 224000, P.R. China; 4 Tongjin Yin Department of Pediatrics, Affiliated Hospital 6 of Nantong University; Pediatrics Department of Yancheng Third People’s Hospital, Yancheng, Jiangsu Province 224000, P.R. China

**Keywords:** Hyperbilirubinemia, Infant, Newborn, Exchange transfusion, Mathematical Computing, Single-volume, Double-volume

## Abstract

**Objective:**

To study the optimal blood exchange transfusion (BET) capacity in neonates with severe hyperbilirubinemia and establish a mathematical standard model for calculating the volume of BET.

**Methods:**

The clinical data of 30 neonates with severe hyperbilirubinemia who received BET in the NICU of Sixth Affiliated Hospital of Nantong University between January, 2016 to December, 2022 were analyzed retrospectively. All neonates received single-volume exchange transfusion (SVET). Serum bilirubin levels were measured every 15 minutes, and serum calcium, blood glucose, hemoglobin, and platelet levels were monitored every 30 minutes of the procedure. The observation decline curve of bilirubin was plotted, and a standard mathematical model for the arteriovenous synchronous exchange transfusion was established. The standard control curve was then calculated, and the two curves were analyzed and compared. The incidence of adverse events was compared between the first and the second half of BET.

**Results:**

There was a statistically significant difference in hemoglobin levels and platelet counts between different points of the procedure (0, 30, 60, 90, and 120 minutes; F = 12.710, 68.620; P < 0.001). During exchange transfusion, there was a significant decline in bilirubin levels every 30 minutes (F = 5273.00, P < 0.05). The clearance of total serum bilirubin (TSB) in the first half of the BET was higher, and the incidence rate of major adverse events was significantly lower than in the second half (P < 0.05).

**Conclusions:**

SVET can effectively reduce total serum bilirubin levels in neonates with severe hyperbilirubinemia. Clinicians may use SVET for treating the condition in combination with phototherapy.

## INTRODUCTION

Hyperbilirubinemia (HB), which affects 1.1 million infants worldwide each year, is a common neonatal disease that is considered a main cause of rehospitalization.^1^,^2^ Severe hyperbilirubinemia can lead to bilirubin encephalopathy and nuclear jaundice, and numerous complications such as hearing loss, cerebral palsy, and mental retardation that may seriously impact healthy growth and development.^3^ Exchange transfusion, which can rapidly reduce circulating bilirubin levels, is considered the most effective treatment for severe hyperbilirubinemia.^4^,^5^

Double-volume exchange transfusion (DVET), which involves replacing the complete neonatal blood volume twice with blood from an adult donor, is commonly used in newborns with severe jaundice.^6^ A clinical trial by Amato et al^7^ showed that the effectiveness of single volume exchange transfusion (SVET) for treatment of full-term infants with jaundice due to AB0 incompatibility is at least comparable to that of DVET. Later, a systematic review by Thayyil et al^8^ comparing SVET with DVET in jaundiced newborn infants reported that there was insufficient evidence to support or refute the use of single-volume as opposed to double-volume exchange transfusion in jaundiced newborns, as the therapeutic effect of the blood exchange transfusion (BET) varies significantly due to the differences in methods, the speed of blood exchange, and the selection of blood source.^9^ However, a recent study by Xiong et al^10^ suggested that SVET may be more beneficial than DVET, as it has a lower impact on the internal environment of newborns and is associated with shorter exchange times while still achieving the purpose of clinical treatment. Despite this, the evidence of the benefits of SVET over DVET remains sparse.

In our clinical practice of BET, we observed that the continuous dynamic decline in serum bilirubin concentration was most pronounced during the first half of the procedure and significantly diminished in the second half. In addition, no previous studies have combined empirical bilirubin-decline data with a mechanistic model to optimize exchange transfusion volume and timing. Therefore, this study aimed to explore the feasibility of single-volume exchange transfusion therapy in neonates with severe HB and develop a standard mathematical model for calculating the volume of BET.

## METHODS

This retrospective study was conducted in the neonatal intensive care unit (NICU) of Sixth Affiliated Hospital of Nantong University (Jiangsu, China) from January 2016 to December 2022 and included complete records of 30 neonates with severe HB that underwent BET. The causes for HB were hemolysis due to ABO incompatibility. The standard for blood exchange was determined according to the guidelines for the diagnosis and treatment of the newborn’s yellow fever by the American Pediatric Association.^11^

### Ethical approval:

This study was approved by the ethics committee of Yancheng Third People’s Hospital, No. 2025-02, date: January 13, 2025.

### Inclusion criteria:

- Gestational age ≥35w.
- Birth weight≥2500g.
- Total serum bilirubin ≥427umol/l.4.
- Underwent BET.

### Exclusion Criteria:

- Cases of unexplained HB were excluded from the study.
- Neonates who were unable to complete the exchange transfusion within two hours due to catheter issues or who had major congenital anomalies.

### Procedure:

Fresh red blood cell (RBC) suspension was used for exchange transfusion. For neonates with hemolytic disease caused by ABO incompatibility, recombinant blood consisting of type O RBCs and type AB plasma was selected. For those with hemolytic disease due to Rh incompatibility, blood was selected based on Rh compatibility with the mother and ABO compatibility with the neonate. In other cases of severe hyperbilirubinemia, type-specific RBCs and plasma were used. The RBC/plasma ratio was 2:1, and the transfusion volume was 160/kg. Strict cross-matching tests were conducted before blood exchange. Synchronous continuous blood exchange of arterio-venous components was controlled by a volumetric pump.

A peripheral arterial line (blood out) was established in the radial, brachial, or femoral artery using an indwelling needle puncture. Heparin anticoagulation (1 U/mL heparin, 30 mL/h) was administered using a 3-way stopcock system. The total exchange rate (blood out) was 270 mL/h; the volume of blood out was calculated using a measuring cup. The contralateral peripheral vein was used to establish a line, and RBCs and plasma were transfused at rates of 160 mL/h and 80 mL/h, respectively. All paths were closed-loop operations, with flow rates controlled by a computerized infusion pump. The total exchange duration was set to 120 minutes. Cases in which exchange transfusion could not be completed within 120 minutes due to catheter blockage or other complications were excluded from the study.

### Collected indicators:

The level of serum bilirubin was measured every 15 minutes, and the levels of hemoglobin, platelets, serum calcium, and blood glucose were measured every 30 minutes. Bilirubin conversion rate = (bilirubin value before transfusion-bilirubin value at different time points) / bilirubin value before transfusion×100%.

### Establishment of a standard control mathematical model:

Assuming an idealized neonatal exchange transfusion model, the total vascular blood volume of the neonate is denoted as *V* (L), with an initial bilirubin concentration of *c* (μmol/L) at time *t* (min). The exchange transfusion is performed at a continuous rate of *k* (mL/min). Assuming that the incoming blood contains negligible bilirubin and that it mixes uniformly with the patient’s blood, the dynamic bilirubin concentration can be described by the general solution of the differential equation: *b=c×e^(-kt/V)^*, where *b* (μmol/L) represents the bilirubin concentration at time *t*, and *e* is the base of the natural logarithm. Based on this mathematical model, by setting the initial bilirubin concentration, exchange transfusion rate, and total vascular blood volume, a standard bilirubin decline curve can be computed.

Furthermore, assuming the exchange transfusion is completed within 120 minutes, and the total exchange volume is twice the vascular blood volume (i.e., 2V), the exchange rate is determined as k = (2V/120) *mL/min*. Substituting this into the general solution simplifies the equation to *b=c×e^(-t/60)^*, which allows for the calculation of bilirubin concentration at any given time point.

### Adverse events:

Platelets less than 100×10^9^/L; hypocalcemia: serum calcium concentration <1.8 mmol/L or ionized calcium concentration <1 mmol/L; blood glucose <2.8 mmol/L; hypotension: systolic blood pressure <40 mmHg; postoperative complications of infection; transfusion-related allergic reactions; heart rate >180 beats/min for non-crying reasons, etc.; decrease in oxygen saturation <85%; transfusion-related deaths.

### Statistical analysis:

All the test data were processed by prism6 software, and the measurement data conforming to normal distribution were expressed as indicated, and the non-normally distributed ones were described as *M (Q1, Q3)*, and were analyzed by ANOVA with one-way repeated measures; the count data were expressed as percentages, and were tested by χ^2^ test or fisher’s exact test, and the difference was considered statistically significant with P<0.05.

## RESULTS

A total of 30 neonates were included. The average age of all neonates was 5.4±1.6 days, with an average weight of 3520±352g, and a gestational age of 38.4±1.7 weeks. Table-I. The time between admission to the hospital and exchange transfusion therapy was 4.5 (2.5 ± 6.7) hours. The causes of severe hyperbilirubinemia were: ABO hemolytic disease (n=22), anti-D hemolysis of the Rh blood group (n=2), anti-C and E hemolysis (n=1 each), subdural hematoma (n=2) and two cases of unknown etiology.

**Table-I:** Baseline characteristics of the study population.

Parameter	n=30
Age at admission (days), median (1st and 3rd quartile)	4(1,5)
Birth weight (g), mean±SD	3520±352
Gestation (weeks), mean±SD	38.4±1.7
Time after admission to blood exchange treatment (hours), median (1st and 3rd quartile)	4.5(2.5, 6.7)
Male sex, n (%)	17(56.7%)
Mean bilirubin concentration at admission(μmol/L), mean±SD	498.1±37.6
Acute bilirubin encephalopathy, n(%)	1(3.3%)
***The etiology of hyperbilirubinemia, n(%)*** ABO incompatibility Other reasons	26(86.7) 4(13.3)

### Changes in the levels of serum indices:

There was a significant overall difference in the levels of hemoglobin and platelet counts at each time point during BET (t=12.7, 68.6; P<0.05). Hemoglobin and platelet count at the end of BET (120 min) were significantly lower than before the procedure (F = 68.6, P < 0.05). Blood calcium and blood glucose tests before and after the transfusion were comparable Table-II.

**Table-II:** Comparison of changes in hemoglobin concentration, platelet count, serum calcium and blood glucose concentration at different time points after exchange transfusion (*χ̅*±*S*).

Time point	Hemoglobin (g/L)	Platelet counts (×10^9^/L)	Blood calcium (umol/l)	Blood glucose (umol/l)
0min	136.4±15.2	251.4±50.6	2.08±0.15	4.2±1.2
30min	131.7±17.2	233.7±49.8	2.05±0.12	3.9±0.8
60min	120.8±12.5	205.2±45.2	1.99±0.12	3.8±0.7
90min	123.6±8.9	142.1±39.9	2.04±0.10	3.9±0.4
120min	115.4±10.4a	99.6±18.31a	2.01±0.13	4.0±1.1
*F*	12.7	68.6	2.36	0.86
*P*	0.00	0.00	0.06	0.48

Compared with pre-BET, ^*^P<0.05, *^#^P>*0.05.

### Time-bilirubin decline curves:

**Fig.1:**
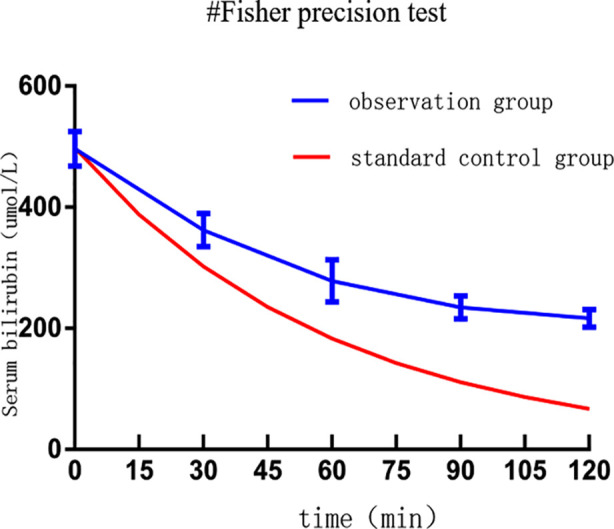
Time-bilirubin decline curves.

The dynamic bilirubin decline curve, measured every 15 minutes, and the standard reference decline curve are shown in Fig.1. The observation decline curve of bilirubin was plotted, and a standard mathematical model for the arteriovenous synchronous exchange transfusion was established. The standard control curve was then calculated.

The observation decline curve of bilirubin demonstrated bilirubin concentrations of 498.1 ± 37.6 μmol/L, 378.5 ± 27.2 μmol/L, 278.4 ± 34.9 μmol/L, 234.6 ± 18.9 μmol/L, and 216.7 ± 26.7 μmol/L at 0 minutes, 30 minutes, 60 minutes, 90 minutes, and 120 minutes, respectively. Overall, there were significant differences in bilirubin concentration at various time points during BET (F=5273.0, P < 0.05). The decline pattern was essentially consistent with the standard reference curve. The slope of the tangent to the decline curve gradually decreased over time. The slope of the tangent of the standard reference slope was steeper than that of the observation group. From 0 to 60 minutes, the decline curve was relatively steep, with a bilirubin removal rate of (42.5 ± 5.4) %. Between 60 and 120 minutes, the decline was considerably lower, with a bilirubin removal rate of 24.3 ± 8.7% (t = 9.740, P < 0.05). At 0 minutes (before blood exchange) and 120 minutes (end of blood exchange), there was a marked decrease in bilirubin concentration, with a statistically significant difference (t = 33.420, P < 0.05).

### Adverse events:

Of a total of 53 adverse events reported during BET, seven occurred within 0-1 hours, and 46 occurred within 1-2 hours, with a statistically significant difference (P < 0.001). No hypoglycemia, blood pressure drops, and fatalities were observed. The most common adverse events included thrombocytopenia, drop in oxygen saturation, increase in heart rate, and transfusion-related anaphylactic reactions (Table-III). Thrombocytopenia was not observed in the first half of the BET, whereas 16 cases were observed in the second half, with a statistically significant difference (P < 0.001). Similarly, the rate of transfusion-related allergic reactions was higher in the second half of the BET (P < 0.05) (Table-III).

**Table-III:** Comparison of (0-1) h and (0-2) h adverse events in the course of blood exchange.

Adverse Events	(0-1) h, n=30 n (%)	(1-2) h, n=30, n (%)	χ^2^	P
Total	6(20)	40(133.3)	32.2	0.000
Thrombocytopenia	0(0)	16(53.3)	--	0.000#
Hypocalcemia	1(3.3)	0(0)	--	1.000#
Apnea	1(3.3)	3(10)	2.78	0.100
Increased heart rate	2(6.7)	12(40)	9.31	0.010
Transfusion-related allergic reactions	2(6.7)	9(30)	5.45	0.040

#Fisher precision test.

## DISCUSSION

This study aimed to determine the optimal BET capacity in neonates with severe hyperbilirubinemia and establish a mathematical standard model for calculating the volume of transfusion. The results showed that SVET is sufficient to effectively reduce total serum bilirubinemia levels in neonates with severe hyperbilirubinemia. BET is an effective treatment for severe neonatal hyperbilirubinemia, can rapidly reduce plasma bilirubin levels, and replace sensitized red blood cells in patients with hemolytic disease.^12^ BET plays an irreplaceable role in preventing and treating bilirubin-induced hearing loss and bilirubin encephalopathy.^13^,^14^

Currently, reports on BET are limited to the relationship between bilirubin levels and the volume of transfusion and often lack standardized time and speed parameters.^9^,^10^,^15^ The observation decline curve established in this study demonstrated that the bilirubin removal rate in the first half of the BET was significantly higher than in the second half, with nearly half of the bilirubin removed by the SVET. This result suggests that traditional BET could be further optimized and improved to achieve therapeutic goals while conserving blood resources. The study revealed that the dynamic decline in bilirubin exhibits an exponential relationship with the exchange transfusion time, characterized by an accelerated initial decline that gradually slows as time progresses.

The slope of the observation decline curve was highly consistent with that of the standard control curve during the first 60 minutes. However, from 90 to 120 minutes of BET, the curve flattened significantly. This difference between the observation and the ideal standard curve can be attributed to factors such as the continued production of bilirubin during exchange transfusion and the re-diffusion of bilirubin from tissues into the blood after the intravascular bilirubin concentration decreases. The study also demonstrated that the decline in bilirubin concentration is not directly proportional to the volume of exchange. While the calculated bilirubin removal rate of the standard curve reached 63.2% after 60 minutes of BET, the observation group demonstrated 44.1% bilirubin clearance after SVET, with nearly half of the bilirubin removed from the perspective of hyperbilirubinemia intervention standards, these results meet the therapeutic goal.

Previous research confirms the results of this study and reports that the bilirubin removal rate in SVET may range from 37.6% to 58.5%,^15^,^16^ sometimes approaching the effectiveness of DVET.^7^ These fluctuations in bilirubin removal rates are likely related to differences in blood source selection and inconsistencies in the duration of exchange transfusion across various studies. This study demonstrated that, despite the same volume of blood being exchanged during the second half of BET, the decline in bilirubin was limited. Additionally, the adverse events during exchange transfusion occurred more frequently in the second half of the treatment compared to the first half.

Other studies have reported that a two-stage SVET can effectively reduce bilirubin levels.^17^ However, the study lacked data on bilirubin levels at the end of the first stage of treatment. Essentially, it involved two SVETs, which were more efficient than DVET, indirectly proving the effectiveness of single-volume exchange transfusion. It is plausible that the bilirubin-time mathematical model developed in this study can better clarify the association between the removal efficiency and the exchange transfusion speed, as well as the child’s blood pool volume. With SVET, the exchange speed is equal to the blood pool volume divided by 60 minutes. If the exchange is controlled to one hour, it can significantly reduce bilirubin levels. Furthermore, this study showed that the average interval time from hospital admission to the start of BET in all 30 patients was approximately 4.5 hours. Therefore, clinicians may consider incorporating active phototherapy before BET to further improve bilirubin clearance.

In this study, the average platelet count of patients after BET was lower than the normal value, with 16 out of 30 (53.3%) neonates presenting with platelet counts below the lower normal limit at the end of the transfusion. Notably, the decline in the platelet count occurred during the second half of the treatment. Therefore, SVET has a significantly lower impact on platelet count compared to DVET, which is consistent with previous reports.^9^,^18^

Hypocalcemia is a common adverse event during BET and often requires treatment.^19^–^21^ Studies suggest that the occurrence of hypocalcemia is related to the citrate anticoagulant in red blood cell suspension.^22^ However, whether routine calcium supplementation during the exchange transfusion is necessary remains controversial.^23^ In this study, routine calcium supplementation was not administered. Throughout the procedure, the average serum calcium level decreased compared to pre-exchange levels, but the difference was not statistically significant. Only one case of hypocalcemia was observed, which occurred during the first half of the treatment. No calcium supplementation was administered, and no clinical signs of hypocalcemic convulsions were noted. The incidence of hypocalcemia in this study was different from that reported by Chen et al.,^24^ which may be attributed to the fact that all included patients were full-term infants and the various factors related to the blood source selection for BET.

The incidence of transfusion-related allergic reactions and tachycardia in this study was significantly higher in the second half of BET than in the first half. No serious adverse events, such as death or hypotension, were observed. Although the sample size was small, these results further suggest that SVET has a clear safety advantage over DVET.

There was a significant decrease in hemoglobin levels after the full-course exchange transfusion with recombinant blood. This decrease can be attributed to the lack of a standardized hematocrit for red blood cell suspension in China, as well as differences in red blood cell preservation solutions. The hematocrit of red blood cells is typically around 50%.^25^,^26^ Still, with recombinant blood containing plasma, the hematocrit is often significantly lower than 50%, which directly leads to a noticeable drop in hemoglobin levels after the exchange transfusion. Therefore, if the exchange transfusion is completed at the halfway point, the impact can be significantly reduced.

### Limitations:

This study has several limitations. First of all, it is a retrospective single-center study with a small sample size which may have potential unmeasured confounders. Secondly, there is a lack of a contemporaneous DVET control group. Lastly, the rebound bilirubin levels after the exchange transfusion were not statistically analyzed further, and only a theoretical feasibility analysis was conducted using the mathematical model. Prospective multicenter trials should address these gaps.

## CONCLUSION

SVET can effectively reduce total serum bilirubin levels in neonates with severe hyperbilirubinemia. Clinicians may use SVET for treating the condition in combination with phototherapy.

### Authors’ contributions:

**SC:** Literature search, study design and manuscript writing.

**JP, BW and TY:** Data collection, data analysis and interpretation. Critical Review.

**SC:** Manuscript revision and validation and is responsible for the integrity of the study.

All authors have read and approved the final manuscript.
